# Heat Loss May Explain Bill Size Differences between Birds Occupying Different Habitats

**DOI:** 10.1371/journal.pone.0040933

**Published:** 2012-07-25

**Authors:** Russell Greenberg, Viviana Cadena, Raymond M. Danner, Glenn Tattersall

**Affiliations:** 1 Smithsonian Migratory Bird Center, Smithsonian Conservation Biology Institute, National Zoological Park, Washington, DC, United States of America; 2 Department of Biological Sciences, Brock University, St. Catharines, Ontario, Canada; 3 Avian Ecology Group, Department of Biological Sciences, Virginia Polytechnic Institute and State University, Blacksburg, Virginia, United States of America; University of Lethbridge, Canada

## Abstract

**Background:**

Research on variation in bill morphology has focused on the role of diet. Bills have other functions, however, including a role in heat and water balance. The role of the bill in heat loss may be particularly important in birds where water is limiting. Song sparrows localized in coastal dunes and salt marsh edge (*Melospiza melodia atlantica*) are similar in size to, but have bills with a 17% greater surface area than, those that live in mesic habitats (*M. m. melodia*), a pattern shared with other coastal sparrows. We tested the hypotheses that sparrows can use their bills to dissipate “dry” heat, and that heat loss from the bill is higher in *M. m. atlantica* than *M. m. melodia*, which would indicate a role of heat loss and water conservation in selection for bill size.

**Methodology/Principal Findings:**

Bill, tarsus, and body surface temperatures were measured using thermal imaging of sparrows exposed to temperatures from 15–37°C and combined with surface area and physical modeling to estimate the contribution of each body part to total heat loss. Song sparrow bills averaged 5–10°C hotter than ambient. The bill of *M. m atlantica* dissipated up to 33% more heat and 38% greater proportion of total heat than that of *M. m. melodia*. This could potentially reduce water loss requirements by approximately 7.7%.

**Conclusions/Significance:**

This >30% higher heat loss in the bill of *M. m. atlantica* is independent of evaporative water loss and thus could play an important role in the water balance of sparrows occupying the hot and exposed dune/salt marsh environments during the summer. Heat loss capacity and water conservation could play an important role in the selection for bill size differences between bird populations and should be considered along with trophic adaptations when studying variation in bill size.

## Introduction

The avian bill is iconic for how evolution shapes morphology in response to changing environments. Variation within and between bird species’ bills conventionally has been interpreted in light of differences in foraging behavior and diet, and studies of the avian bill provide some of the strongest evidence of the effects of food supply on a morphological feature. Indeed, the shape of bills between closely-related species or subspecies has been shown to relate to forage and dietary differences [1]–[5]. Some studies have gone further and determined the functional basis for differences in bill morphology that relate to differences in diet [1], [6]–[10]. Short-term changes in bill size have been associated with climate-induced changes in resource availability [7] and the addition and subtraction of potentially competitive species [4]. Studies of adaptive radiation and rapid evolution in bill size have become textbook examples of the evolution of ecologically important traits.

The avian bill may also play an important and heretofore under-appreciated role in body temperature regulation. Appendages and other extremities may be a source of particularly intense heat loss and thus a reduction in size will conserve, and an increase will dissipate, a disproportional amount of heat [11], [12]. Some older papers presented data suggesting a correlation between temperature and bill size within some avian species (*e.g.,* [13]), with larger bills occurring in warmer climates. Symonds *et al*. [14] analyzed bill size in over two hundred species in several orders of non-migratory birds and found that bill size decreases with low minimum temperature. VanderWerf [15] further documented such a pattern within the elepaios (*Chasiempis* spp.).

Until recently, however, the possible ecological importance of the bill in thermal balance has not been the focus of much research, possibly because of a belief that the bill is insufficiently vascularized and of too small a size [16], [17] to be an important source of heat loss compared to other appendages (such as flippers and ear pinnae of mammals). It is well known, however, that bills grow and wear continuously [18] and that the tissue within the ramphotheca (horny bill covering) is vascularized [19], [20]. Furthermore, recent thermographic research has revealed that heat loss from bills can be substantial, at least in larger birds [21]–[23].

If bill size is selected for heat dissipation or conservation, then this opens the question of what process during which season drives the evolution of bill size. Although bill size is known to vary seasonally in many birds [18], [24], the magnitude of this variation is quite small. So a single bill size might only be able to maximize heat dissipation or minimize heat loss (for a discussion of this trade-off for avian tarsi see [25]). Historically, the relationship between climate and bill size has focused on heat conservation [13], [14], with the argument that smaller bills evolve in areas with colder winters. Speakman and Król [26] advocated a greater focus on heat dissipation during periods of resource flush, as opposed to energy acquisition and conservation during resource poor seasons, when attempting to understand adaptations in endotherms. This approach leads to the hypothesis that increased bill size facilitates the loss of excess metabolically produced heat under hot conditions. Scholander [27] dismissed the importance of Allen’s rule because of the well-known ability of many larger animals to constrict blood flow to extremities and limit heat loss during periods of cold ambient temperature. By this argument, if birds are indeed able to reduce blood flow to the bill without constraints, then a large bill could evolve to maximize heat dissipation during periods of high temperature with little cost during cold periods. Consistent with this idea, Tattersall *et al*. [23] demonstrated that adult Toco toucan (*Ramphastos toco*) were able to shunt blood flow into the tissue underlying the ramphotheca during exposure to high temperatures and reduce flow in the face of cold temperatures.

It is unclear how the studies of heat dissipation, focused on a few desultory taxa, are relevant to all birds. Speakman and Król [26] argued that because of surface to volume ratios, the effects of heat dissipation would be revealed primarily in endothermic animals with large core body size and it is among them where we should expect to find increased heat loss from enlarged extremities. Given Speakman and Krol’s [26] emphasis on heat dissipation in large bodied endotherms, it is logical that the documentation of bill vasodilation and heat dissipation has been, thus far, restricted to relatively large-bodied birds.

Heat loss per se, may not be as much of an issue in smaller endotherms [26], but water loss due to evaporation from the skin and respiratory tract can be very high in small endotherms with high metabolic rates, such as passerines [28]–[30]. Thus, although small birds are less sensitive to thermal stress than larger birds, because of their high surface-to-volume ratio, small birds process much more water on a mass-specific basis than do larger birds. Combined with their large relative surface area, this makes them particularly susceptible to the negative impacts of water loss [30]. Evaporative water loss does not completely eliminate excess metabolic heat when birds experience ambient temperatures above their thermal neutral zone but below their body temperature [31], and the heat that is eliminated through evaporation may come at the expense of overall dehydration. Therefore, alternative ways to dump “dry” heat that do not involve water loss should be advantageous.

If the avian bill can be used as a radiator to expel excess heat under thermally stressful conditions, this function should be particularly important in birds that occupy climatically poorly-buffered habitats where opportunities to escape direct insolation are reduced and freshwater is limited. Larger bill size would allow for the release of a greater amount of “dry heat.” Coastal salt marsh and dune-strand habitats are prime examples of environments where birds might experience thermal and water stress. Greenberg and Olsen [32] showed that eleven taxa subspecies or species of tidal marsh sparrows have larger bills than their sister taxa and Greenberg *et al.* [24] presented a striking correlation between high summer temperature and bill size (corrected for body size variation) among and within species of salt marsh dwelling sparrows (Emberizinae).

**Figure 1:**
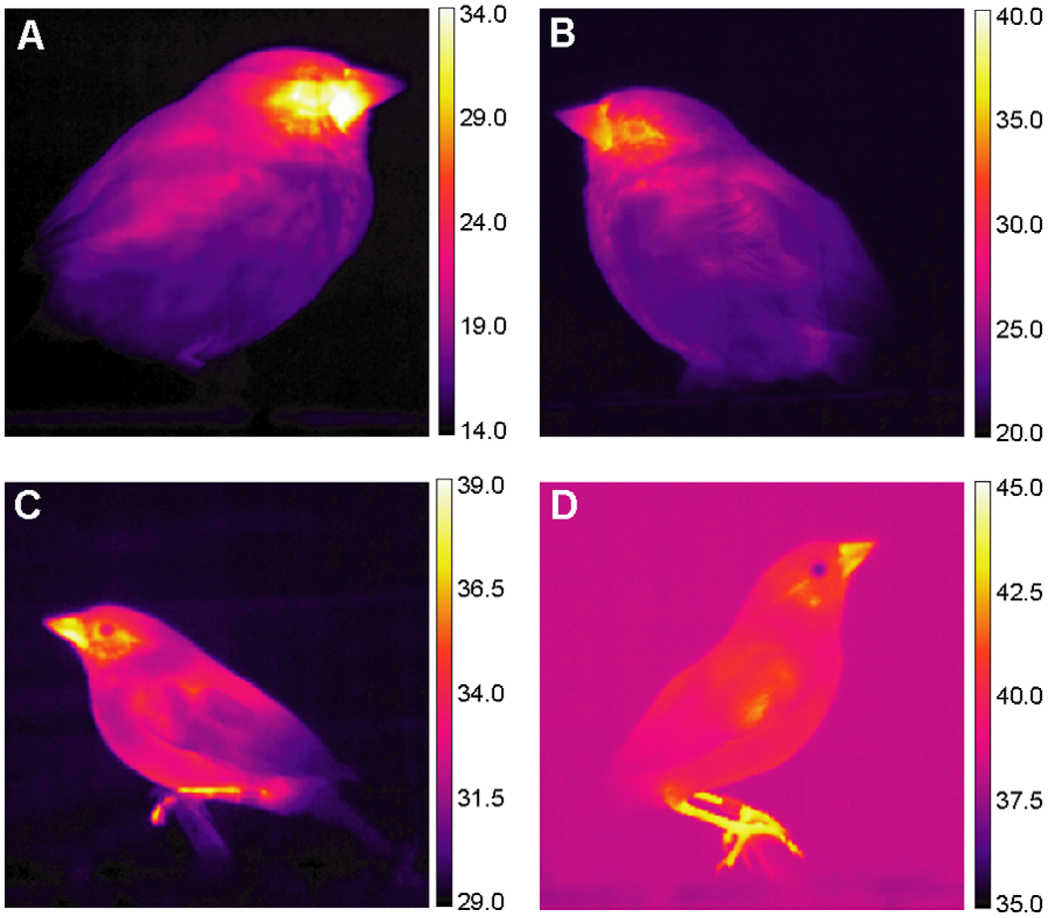
Infrared images of eastern and Atlantic song sparrows. Eastern (A) and Atlantic (B–D) song sparrows were imaged at ambient temperatures of 15 (A), 21 (B), 29 (C), and 37°C (D).

To further evaluate the hypothesis that heat dissipation is driving the evolution of bill size in coastal sparrows, data on the thermal properties of the bill must be acquired to support the assumptions of argument, that a significant amount of heat is lost from the bills and that this varies with bill size. In this study, we compared two subspecies of the song sparrow (*Melospiza melodia*), which occupy thermally divergent habitats in eastern North America; the Atlantic song sparrow (*M. m. atlantica*) breeds in dune scrub and salt marsh edges along the mid-Atlantic coast, whereas the eastern song sparrow (*M. m. melodia*) is widespread in urban and suburban gardens and mesic secondary scrub. Using thermal imaging (see Fig. 1 for sample images) under temperature controlled conditions, we addressed the following four questions: 1) Do small birds maintain bill temperatures above ambient? If so, 2) Does the elevated bill temperature lead to a significant amount of heat loss from the bill relative to the entire heat budget? 3) Does the sparrow subspecies in the more thermally stressful and moisture limited environment release more heat from its bill than the subspecies from the more buffered and mesic environment? And 4) Is heat loss temperature dependent, increasing at higher temperatures, and is there evidence of a threshold transition temperature between vasoconstriction and vasodilation?

## Results

### Morphological Measurements

The only significant subspecies difference was in bill size; Atlantic song sparrows averaged 16.7% greater total surface area (Table 1). Bill cone surface area was also significantly greater in the Atlantic song sparrows (13.1%). The mean values (99.81 versus 88.19 mm^2^) were similar to those obtained from much larger samples of Atlantic and eastern song sparrows measured in the field (Greenberg *et al.* unpubl.) where mean bill surface areas (mm^2^) were 95.8±6.5 SD (n  = 81) and 87.0±5.00 (n  = 79), respectively. The bill surface area values for the experimental sparrows corresponded to an estimated 2.05 and 2.38% of total body surface area for eastern and Atlantic song sparrow, respectively (Table 1).

**Table 1:** Mean (and standard deviation) of morphometrics, with P-values based on t-tests (independent samples) for sparrows used in thermal imaging experiments.

Measurement	Eastern song sparrow	Atlantic song sparrow	P-value (t-test)^a^	Percentage increase^b^
Wing Chord (mm)	65.89 (1.19)	63.92 (1.44)	0.060	2.8
Weight (g)	18.89 (1.27)	19.97 (1.05)	0.922	5.8
Tarsus (mm)	20.01 (0.74)	19.85 (0.89)	1.000	0.8
Bill cone area (mm^2^)	88.19 (4.21)	99.89 (19.81)	0.015	13.1
Total bill area (mm^2^)	127.46 (10.41)	148.70 (12.12)	0.011	16.7
Leg area (mm^2^)	240.07 (20.87)	241.94 (20.61)	1.000	0.8
Body area (mm^2^)	5846.04 (149.26)	5852.78 (226.03)	1.000	0.1
Total bill area as a percent of entire area	2.05 (0.18)	2.38 (0.18)	0.015	16.0
Leg area as a percent of entire area	3.86 (0.27)	3.87 (0.31)	1.000	0.4
Body area as a percent of entire area	94.09 (0.29)	93.74 (0.45)	0.728	0.4

a t-tests assume equal variances in both subspecies and Bonferroni corrections were applied.

b ((Larger taxa mean-smaller taxa mean)/smaller taxa mean)*100%.

### Bill Temperature and Heat Loss

**Figure 2:**
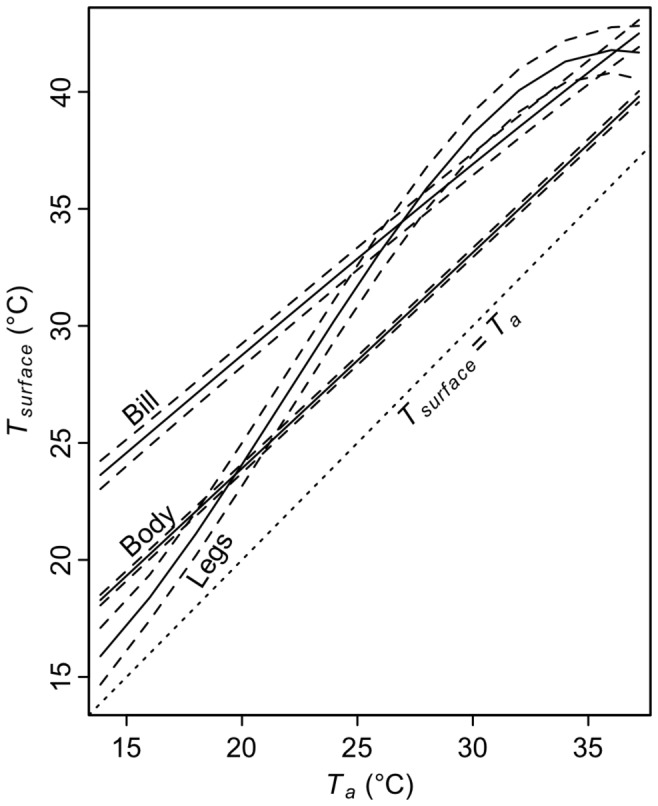
Surface temperature of bill and bill base of song sparrows vs. ambient temperature (*T_a_*). (A) shows values for bill and (B) presents values for bill base. Black lines = model averaged predictions of linear mixed models ± highest and lowest unconditional standard errors. Gray lines = raw data.

**Figure 3:**
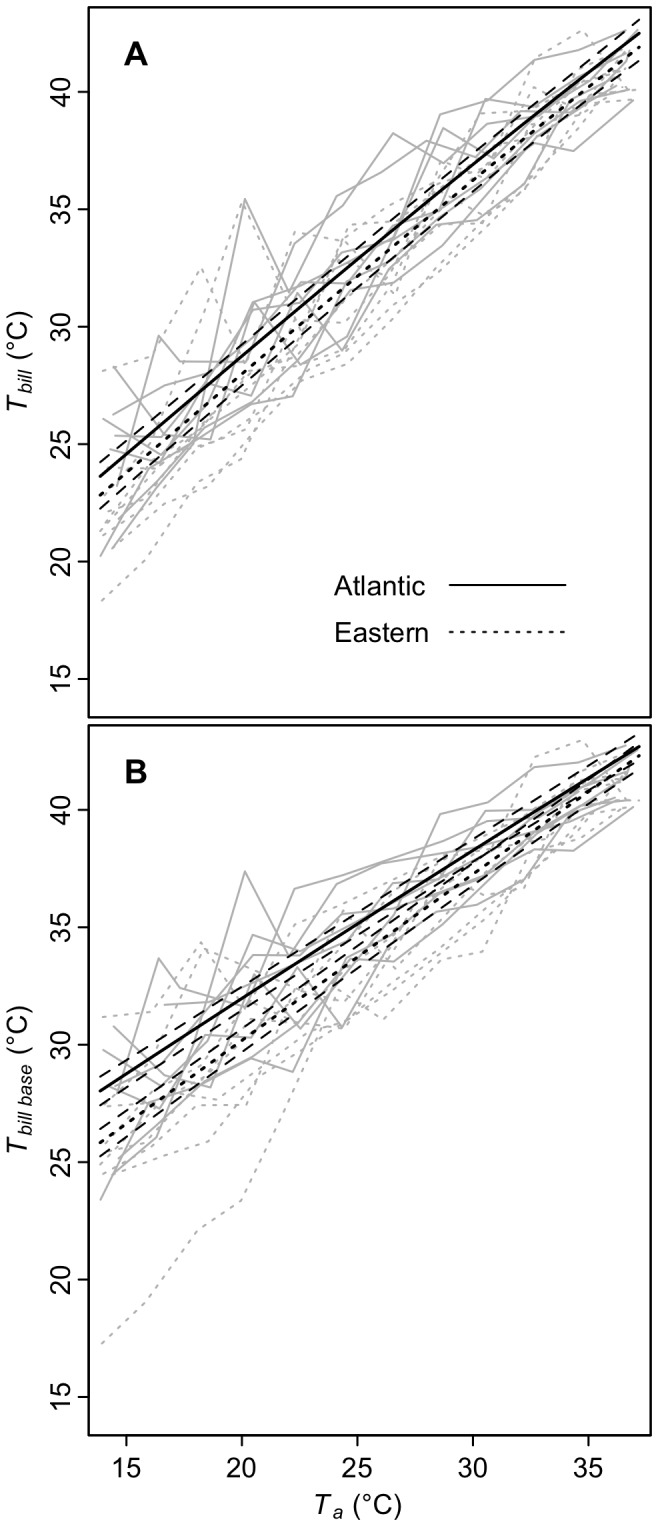
Heat loss through the bill. Total heat loss (*Q_bill_*) is shown in (A) and the percent of total heat loss through the bill (percent *Q_bill_*) is shown in (B). Black lines = model averaged predictions of linear mixed models ± unconditional standard errors. Gray lines = raw data.

**Figure 4:**
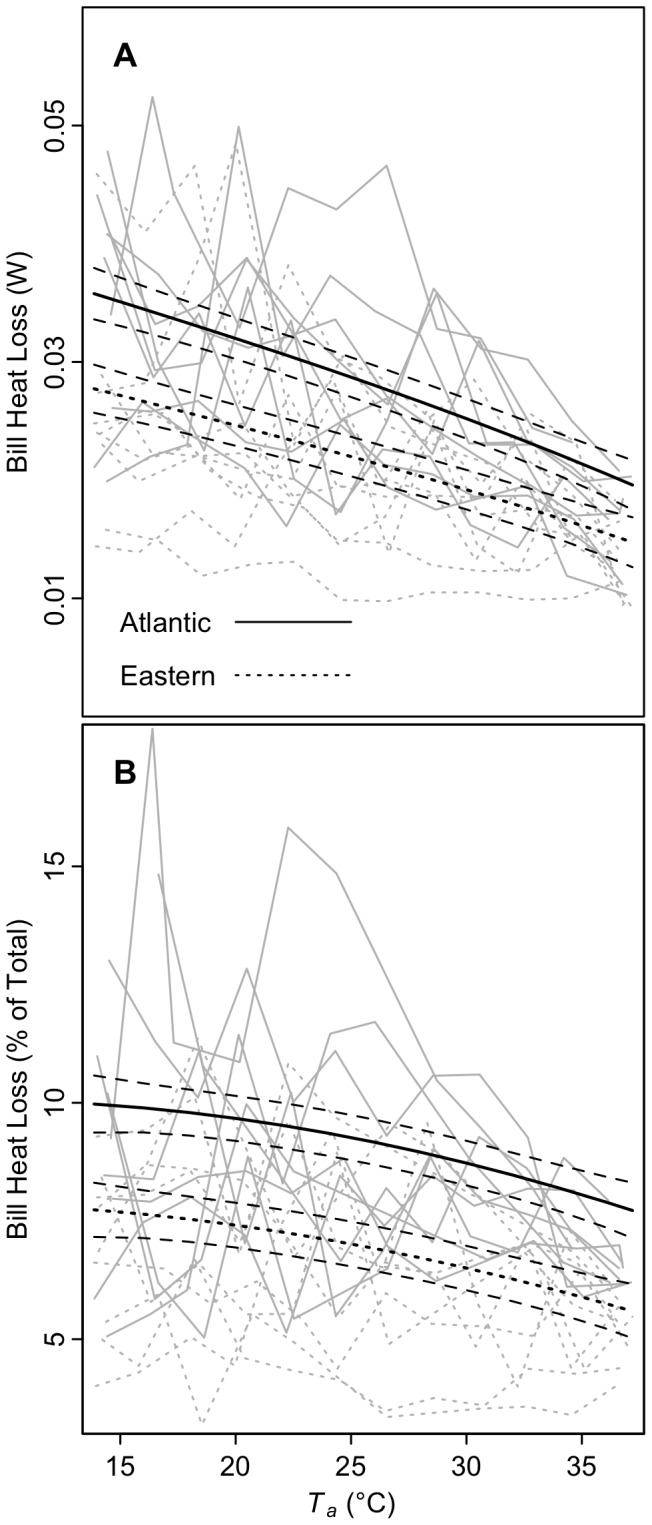
Surface temperatures (*T_surface_*) of the bill, legs (tarsi), and body of both subspecies of song sparrow vs. ambient temperature (*T_a_*). Black lines = model averaged predictions of linear mixed models ± unconditional standard errors. Body and leg surface temperatures did not differ between subspecies (see text). For bill, only the Atlantic subspecies is shown. Gray dashed line indicates *T_a_* = *T_surface_*.

Comparison of fit of linear mixed models revealed that both subspecies maintained surface temperatures of the overall bill (*T_bill_*) and the bill base (*T_base_*) above ambient (*T_a_*) and that *T_bill_* and *T_base_* increased with T_a_ (Fig. 2; Tables 2, 3, Table S3), which resulted in heat loss from the bill (Fig. 3, Tables 4, 5). The relationships between both *T_bill_* and *T_base_* to *T_a_* were close to linear for both subspecies. There was strong evidence that *T_base_* was higher in Atlantic song sparrows, particularly at lower temperatures; the model describing an interaction between subspecies and *T_a_* was the highest ranked, and according to the evidence ratio (calculated from AICc model weights), was 51 times more likely to be the best model in the set than the highest ranked model without a subspecies term (*T_a_*, Table S2). The top model was 17 times more likely than models without interactions, providing support for an interaction between subspecies and *T_a_*. Although subspecies was included in the top model for overall bill temperature, the greater bill temperature found in the Atlantic song sparrow was not well supported (evidence ratio = 1.5). Model average predictions show that song sparrow bills were maintained at 4.7–9.8°C over ambient temperatures within the T*_a_* range of the experiment (15–37°C, Fig. 4). In contrast, body surface temperature was 2.6–4.4°C over ambient and leg temperature increased non-linearly relative to ambient temperature (Fig. 3); neither differed by subspecies (evidence ratios for subspecies = 1.4 and 1.2, respectively, Tables S5 and S6).

**Table 2:** Linear mixed models describing the surface temperature of the bill (*T_bill_*).

Models	K	AICc	ΔAICc	AICc weight
SSP + *T_a_*	6	861.232	0	0.208
SSP *+ T_a_* + *T_a_* ^2^	7	861.571	0.339	0.176
SSP * *T_a_*	7	861.730	0.498	0.162
*T_a_*	5	862.071	0.839	0.137
*T_a_* + *T_a_* ^2^	6	862.371	1.139	0.118
SSP + *T_a_* + *T_a_* ^2^ + *T_a_* ^3^	8	863.079	1.847	0.083
*T_a_* + *T_a_* ^2^ + *T_a_* ^3^	7	863.884	2.652	0.055
SSP * *T_a_* + SSP * *T_a_* ^2^	9	864.018	2.786	0.052

Individual is a random effect and square root of activity is a fixed effect in each model. SSP = subspecies, *T_a_* = ambient temperature. Models with ΔAICc <5 are included here (see full model set in Table S1).

**Table 3:** Linear mixed models describing surface temperature of the bill base (*T_base_*).

Models	K	AICc	ΔAICc	AICc weight
SSP * *T_a_*	7	860.717	0	0.720
SSP * *T_a_* + SSP * *T_a_* ^2^	9	863.740	3.024	0.159

Individual is a random effect and square root of activity is a fixed effect in each model. Models with ΔAICc <5 are included here (see full model set in Table S2).

**Table 4:** Linear mixed models describing heat loss through the bill (*Q_bill_*).

Models	K	AICc	ΔAICc	AICc weight
SSP * *T_a_*	7	−1578.213	0	0.462
SSP + *T_a_*	6	−1576.144	2.070	0.164
SSP * *T_a_* + SSP * *T_a_* ^2^	9	−1575.855	2.358	0.142
SSP + *T_a_* + *T_a_* ^2^	7	−1575.697	2.517	0.131
SSP + *T_a_* + *T_a_* ^2^+ *T_a_* ^3^	8	−1574.063	4.150	0.058

Individual is a random effect and square root of activity is a fixed effect in each model. Models with ΔAICc <5 are included here (see full model set in Table S3).

**Table 5:** Linear mixed models describing heat loss through the bill as a percent of heat of heat lost through all body surfaces (percent *Q_bill_*).

Models	K	AICc	ΔAICc	AICc weight
SSP + *T_a_* + *T_a_* ^2^	7	784.714	0	0.358
SSP + *T_a_*	6	785.129	0.416	0.291
SSP * *T_a_*	7	786.828	2.114	0.124
SSP + *T_a_* + *T_a_* ^2^+ *T_a_* ^3^	8	786.860	2.147	0.122
SSP * *T_a_* + SSP * *T_a_* ^2^	9	787.644	2.930	0.083

Individual is a random effect and square root of activity is a fixed effect in each model. Models with ΔAICc <5 are included here (see full model set in Table S4).

Heat loss through the bill was greater in Atlantic song sparrows than eastern song sparrows, both absolutely (*Q_bill_* heat loss, Fig. 3A, Table 4, evidence ratio = 50) and in relation to the rest of the body (percent *Q_bill_*, Fig. 3B, Tables 5 and S4, evidence ratio = 39). Model average predictions show that, in comparison to eastern song sparrows, Atlantic song sparrows dissipated 29–33% more heat through the bill and 29–38% more heat through the bill as a proportion of total heat lost.

## Discussion

These experiments extend the avian taxa that show evidence of vascular dilation in the bill from a few large bodied birds [21]–[23] to small birds. Song sparrows have substantially elevated bill temperatures averaging 4.7–9.8°C over the range of ambient temperatures presented. The bill accounted for 5.6–10% of the total heat loss in the two subspecies of song sparrow despite of only making up 2.05 and 2.38% of total surface area.

The larger bill size and possibly higher bill temperature result in Atlantic song sparrows losing approximately 33% more heat from its bill than the eastern song sparrow. Taken together with previous findings that coastal salt marsh sparrows have larger bills than their sister taxa [32], [33], coastal marsh sparrow taxa that experience hotter summers have larger bills [24], and bill surface area of *M. melodia* in California is correlated to summer high temperatures (Greenberg and Danner unpub. data), the greater heat loss in the Atlantic sparrow provides further evidence that larger bills are selected to dissipate more heat in these thermally stressful and fresh-water limited environments.

Most of the difference in heat dissipation between subspecies can be attributed to the size of the bill. It is unclear from these experiments the degree to which other factors, such as the degree of vasodilation, contributes as well. A greater temperature at the base of the bill of the Atlantic song sparrow is well supported (a model with the interaction between T*_a_* and subspecies was 51 times more likely than the top model without subspecies), whereas a greater overall bill temperature has very weak support. The largest blood vessels and greatest blood volume is probably found at the base of the bill (see [20]). We may have detected a well-supported difference in the temperature of the base and not the entire bill because, given that the base is the area with the greatest vasculatization and heat loss, it may be easier to develop an estimate of the effect of size given the amount of individual variation. Evaluating the existence of a difference in overall bill temperature between the subspecies will require further sampling, but such a difference would be an additional avenue for adaptation to thermal environments and thus deserves further investigation. The important point, established in this study, is that an increase in bill size within song sparrows leads to greater heat dissipation.

The amount of heat dissipation from the larger bill of the Atlantic song sparrow should be viewed in terms of marginal values. There is a fixed amount of heat and water that will be lost from all song sparrows. A particular population of song sparrows expanding into a thermally challenging environment will only be able to increase, marginally, from this base value. The expansion of the bill surface needs to be evaluated against other options for marginal increase in heat loss and water conserved. The bill and tarsi are uninsulated and because of their keratin covering, impermeable to evaporative water loss, and hence qualitatively more effective vehicles for dry heat dissipation than other possible surfaces. It is possible that due to constraints in counter current exchange of blood flow and the frequency with which they are covered by contour feathers, that tarsi are less important in heat exchange [14] than the bill. To illustrate the potential importance of the difference in heat loss from the bill to the water budgets we offer the following calculations. The 33% difference in heat loss from the bill between the Atlantic and eastern song sparrows corresponds to 6.5 mW of dry heat loss difference. The potential water savings afforded by this would (using a latent heat of vaporization of 2418 J/g water; [34]) be 9.7 mg/h, which corresponds to approximately 7.7% of the evaporative water loss of a similarly sized sparrow at 25°C [28].

The increment of heat loss will be important if it further affects some other aspect of the ecology of sparrows that increases fitness. In this case, the increase in bill surface area and heat loss may not cause a measurable decrease in mortality of sparrows, but it may allow an individual to remain active during warmer conditions, thus, longer in the day and later in the breeding season. Provisioning pairs could spend more time gathering food and males can increase their territorial defense and mate-guarding behaviors over more thermally stressed neighbors. It may allow more singing during warmer conditions, which is a large source of respiratory water loss because metabolic rate is increased [35] and respired air during singing bypasses the respiratory turbinates, thus potentially providing the larger billed male with a slight edge over its competitors [24]. As long as the cost of increased bill size is lower than these potential benefits, natural selection could favor the larger billed bird even if the fitness advantages are relatively small.

The monotonic decrease of bill heat loss with increasing ambient temperature (Figure 3A) in both subspecies of song sparrows suggests that the bills of the song sparrows are vasodilated throughout the experimental ambient temperature range. The apparent lack of a distinct vasodilation threshold resulted in a shallow decline in heat loss with increasing temperature caused by the reduced heat transfer potential that occurs when the air and the bill temperatures converge (see [36]). The bills of the song sparrows performed similarly to the immature toucans of the Tattersall *et al.* [23] study and unlike the adults, which displayed the ability to reduce blood flow to the bill at lower temperatures. In fact, the bills of the sparrows performed unlike the tarsi, which showed a thermal pattern consistent with a constriction/dilation threshold, which is common to many appendages of endothermic animals [21], [37]–[39].

The ecological significance of this pattern is that heat dissipation from the bill becomes a potential cost at lower temperatures. This pattern was tested down to 15°C, which is well below the minimum critical temperature of the song sparrow’s thermoneutral zone (23°C; [39], [40]), but should be tested at colder temperatures in the future. If the bill is functioning as a thermal adaptation, then this pattern clearly supports heat dissipation in the summer as the critical process and season. As suggested by Greenberg *et al.* [24], migration could contribute to the importance of heat dissipation over heat conservation, as different coastal sparrow taxa tend to migrate to warmer wintering grounds and minimum winter temperatures tend to converge. Another hypothesis that can be readily addressed is that patterns of vasoconstriction vary seasonally and constriction occurs at low temperatures during the winter months when heat conservation becomes important.

The reason that the bill does not vasoconstrict at lower temperatures, whereas the legs do, may relate to constraints imposed by bill function and structure. The relatively large jaw muscles are proximate to the base of the bill, where a majority of the heat is dissipated, and where larger blood vessels should be located [20]. Constriction of blood flow to the base of the bill may be constrained by the circulatory requirements of the jaw musculature. The bill needs to function in resource acquisition at all temperatures, and in small temperate zone granivores, feeding rates are probably maximal during the coldest conditions. The legs are more distal to any muscle masses, exhibit counter-current blood flow, and may be free to constrict their peripheral shunt vessels, allowing the limbs to act alternatively as both conservators and dissipators of heat [38], [41], [42].

In this study, we compared the heat dissipation by bills of sparrows from two habitats in a controlled environment where some important phenomena, such as radiative heat gain and wind were absent. Future work in the field will be necessary to determine exactly how the convective heat loss we observed in the laboratory functions in the wild. With this caveat, we suggest that the thermographic data provide support for the previously hypothesized mechanism underlying the observation that sparrows in the thermally stressful, fresh-water limited coastal salt marsh/dune habitats evolved larger bills to dissipate dry heat [24]. Little is known about the diet of the Atlantic song sparrow. So clearly, the possibility that the effect of climate on bill size is indirect, with climate acting through food availability and trophic adaptation, needs to be addressed alongside the physiological approach. The same, however, can be said for studies that focus solely on trophic ecology to explain bill size variation. In light of the issues raised by Speakman and Król [26], the evolution of bill size is an excellent system to evaluate the importance of energy consumption/conservation versus heat dissipation. Bill size could be shaped by the ability to gain energy through greater efficiency in foraging, the ability to conserve energy by reducing heat loss, or the ability to dissipate heat. Or, as is more likely the case, bill size is the result of adaptive compromises to combinations of these factors.

## Methods

### Study Species

The song sparrow is one of the most widespread, abundant, and polytypic species of North American birds. Most of the 25 recognized subspecies [43] reside in the western portion of the continent. Most of North America supports the eastern song sparrow (*M. m. melodia*). The Atlantic song sparrow (*M. m. atlantica*) breeds in a narrow strip of sand dune and salt marsh edge habitat along the outer coast from Long Island to the Outer Banks of North Carolina. Although the ambient temperature of the coastal dune/salt marsh habitat may be slightly cooler than inland sites, the habitat is characterized by little shade, high surface temperatures and irradiance levels, and (in the case of the dunes) generally xeric conditions [44], [45].

### Field Collection, Care, and Measurement

Between 15 June and 2 July, 18 male song sparrows were captured by target netting (song playback and a single mist net) and brought to the National Zoological Park, Washington, DC, for thermal imaging experiments. Nine males were captured within the breeding range of the Atlantic song sparrow as determined by morphological analysis of museum study skins and recently captured individuals (Greenberg *et al.* in prep) with six birds obtained at Beach Plum Island, DE (38.81°, −75.19°) and three from Assateague Island National Seashore (38.21°, −75.16°). Nine males were captured within the breeding range of the eastern song sparrow, with four obtained at the National Zoological Park, Washington, DC (38.93°, −77.05°) and five from Takoma Park, MD (38.99°, −77.00°).

Upon capture, birds were placed in paper lunch sacks and transported to the holding facility. Sparrows were kept in small chrome wire finch cages (22 cm×22 cm×22 cm) with two elevated 1 cm diameter dowling perches and a cage floor covered with newspaper. All sparrow cages were placed on metal racks in a single windowless room kept at approximately 25°C and a 13 h∶11 h light-dark cycle. Sparrows were provided with ad libitum commercial wild bird mixture with small seeds, 3–5 small mealworms/day, and *ad libitum* water.

Once during their confinement, we captured and measured morphometrics of the song sparrows. We used digital calipers (0.01 mm precision) to measure wing chord, tarsus length, width, depth (at mid-point) and bill length, depth and width from or at the anterior edge of the nares. These measurements are comparable to those taken from wild-caught birds and are more commonly used in the literature. Additionally, we measured bill length, depth, and width at the base of the bill to obtain a more accurate and complete estimate of bill surface area. Bill cone surface area (comparable to estimates in other studies) measurements were converted to an estimate of surface area of the distal portion of the bill using the following approximate formula for the lateral surface area of a nearly circular elliptical cone ((W+D)/4)*L*π. Total bill surface area used the same formula with measurements taken at the base of the bill. Surface area of the legs (tarsi) was estimated using the formula for an elliptical cylinder, (π (2((w/2)^2^+(d/2)^2^) – 0.5(w-d)^2^)^1/2^) l * 2, where *w* is the width, *d* is the depth and *l* is the length of the tarsus (the feathered tibia was included in the body surface area). Surface area of the body was estimated according to Walsberg and King [17]: A  = 8.11*m^0.67^, where *A* is surface area and *m* is body mass.

### Experimental Procedures

All experiments were performed inside a temperature-controlled environmental room (Environmental Specialties Inc. model 9–28WR Comb). The room was equipped with a one way mirror attached to a small office. This allowed us to observe the birds, monitor their behavior and well being, as well as remotely control the thermal camera (see “Collection and Analysis of Thermographic Data” below) and the temperature of the room without disturbing the birds. Ambient temperature (*T_a_*) and relative humidity were recorded throughout the experiments using a Kestrel 4000 portable weather monitoring system.

We performed one experiment on each individual. On the day of an experiment, a bird was transported inside its home-cage to the environmental room. Immediately following transport, food and water dishes were removed from the cage and replaced with a small dish of water, which was sufficient to last the entire experiment. At the beginning of the experiment, a sliding opaque divider was used to confine the bird to one half of the enclosure with access to only one wooden perch located perpendicular to the front of the cage. The thermal camera was located directly in front, allowing constant observation of the bird when standing on the perch.

Once the experimenter had exited the room, the bird was allowed a minimum of 15 min to habituate to the experimental conditions at 15°C. The bird was then exposed to 2°C step-wise temperature increases from 15 to 37°C, lasting 15 min at each temperature. Thermographic data was collected during the last five minutes of exposure to each temperature to allow the birds 10 min of habituation to the new temperature. At the end of each experiment, the cage divider was removed and the bird was returned to its holding room.

### Collection and Analysis of Thermographic Data

Thermal videos were recorded at 10 frames s^−1^ using a thermal imaging camera (FLIR T-380, FLIR Systems, Inc.) connected to an acquisition program (Examine-R, FLIR Systems, Inc.). Emissivity was assumed to be 0.96 [38]. Five frames were extracted and analyzed from each five-minute video (1 frame each minute), using specialized software (Examine-R, FLIR Systems, Inc.). The five frames were selected at the end of each minute interval unless the image was out of focus or the bird was looking away from the camera; the default was to use the temporally closest frame. Regions of interest were digitally drawn to obtain the average surface temperature of the cone of the bill (*T_cone_*; corresponding to the portion of the bill protruding from the face), the base of the bill (*T_base_*; corresponding to the of the portion of the bill embedded in the face, approximately 5 mm contiguous to the cone), the whole bill (*T_bill_*: *T_cone_* and *T_base_* together; this was the measurement used for calculations of heat loss, below), the tarsi (*T_legs_*; we chose to analyze the warmest leg, toes were not included because they could not be seen in the images), and the body (*T_body_*; corresponding to the surface of the largest portion of the body visible in the image).

### Estimation of Heat Loss

We estimated heat loss in Watts (*Q*) through each major body surface (*Q_bill_*, *Q_legs_* and *Q_body_*), total heat loss (*Q_total_*) by adding values for all body surfaces, and percent *Q_bill_* as *Q_bill_*/*Q_total_*. *Q* of each body part was estimated as described by Tattersall *et al.* [23], using the following equation:
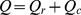
where *Q_r_* is the radiative heat exchange and *Q*_c_ is the convective (forced) heat exchange, such that:



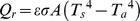


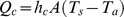
where *ε* is the emissivity for biological tissues (assumed to be 0.96; [38], *σ* is the Stefan-Boltzman constant (5.6703×10^−8^), *A* is the surface area (m^2^) of the body part in question, *T_s_* is the surface temperature of each specific body region, *T_a_* is ambient temperature (°K) and *h_c_* is the heat transfer coefficient:



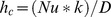

*k* is the thermal conductivity of air at a particular 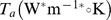.



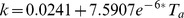



*Nu* is the Nusselt number for each particular body surface.
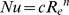
where *c*  = 0.174 and *n*  = 0.618 for the bill and legs values [34], [38] and *c*  = 0.34 and *n*  = 0.6 for the body [46].



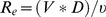
where *V* is the air velocity (assumed to be 0.1 m/s), *υ* is the kinematic viscosity of air at each particular *T_a_*



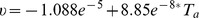
and *D* is the vertical critical dimension for each body part; *l* for legs, *d/2* for bill and volume^1/3^ for body [46]. Volume of each bird was calculated based on mass (*m*) and density (*ρ*) of a sparrow (0.913 g/cm^3^; [47]).



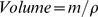



### Data Analysis

To test for effects of *T_a_* and subspecies on *T_bill_*, *T_base_*, *T_body_*, *T_legs_*, *Q_bill_*, and percent *Q_bill_*, we fit linear models to these data with package lme4 [48] in R [49]. Fixed effects of interest included subspecies (SSP) and *T_a_*. To test for potential nonlinear effects caused by vasodilation and decreasing heat transfer potential at higher temperatures, we also fit models including *T_a_* ^2^ and *T_a_* ^3^. To account for repeated sampling of individuals, we modeled individual as a random effect, which allowed each individual to have a different intercept, but constrained all to have the same slope. Because activity (# of hops per minute) had a quadratic relationship with *T_a_* and did not differ between subspecies (see Text S1, Fig. S1), we included the square root of activity in each model as a fixed effect. Correlograms indicated that none of our data sets were temporally autocorrelated [50].

For environmental variables, we used mean values of *T_a_* and relative humidity from the first four minutes of each 5-minute recording period. All environmental values fell within the ranges that both subspecies of song sparrow experience in the wild. Relative humidity during experimentation was negatively correlated to *T_a_* and did not differ between subspecies (evidence ratio of SSP = 0.4, Table S7), so was not included in further analyses. The saturated model in each model set presented here (SSP * *T_a_* + SSP * *T_a_* ^2^ + SSP * *T_a_* ^3^ + activity^1/2^ + random effect of individual) showed homogeneity in the spread of residual values plotted against fitted values, indicating that all models had adequate fit [50].

We determined if subspecies and *T_a_* were important predictors of surface temperature and heat loss by calculating evidence ratios (based on AICc values), which compare the probabilities of models with and without those variables [51]. The evidence ratio is calculated as the weight of the model of interest/the weight of the appropriate null model. To estimate values of each response variable at specific temperature and subspecies combinations, we calculated model averaged predictions and unconditional standard errors (which account for model uncertainty) with the package AICcmodavg [52] in R.

## Supporting Information

Figure S1 **Activity levels vs. ambient temperature (** ***T_a_*** **).** Black line = model predictions for both subspecies combined. Gray lines = raw data.
(TIFF)

Table S1 Linear mixed models describing the surface temperature of the bill (*T_bill_*).
(DOC)

Table S2 Linear mixed models describing surface temperature of the bill base (*T_base_*).
(DOC)

Table S3 Linear mixed models describing heat loss through the bill (*Q_bill_*).
(DOC)

Table S4 Linear mixed models describing heat loss through the bill as a percent of heat of heat lost through all body surfaces (percent *Q_bill_*).
(DOC)

Table S5 Linear mixed models describing the surface temperature of the body (*T_body_*).
(DOC)

Table S6 Linear mixed models describing the surface temperature of the legs (*T_legs_*).
(DOC)

Table S7 Linear mixed models describing relative humidity.
(DOC)

Text S1 Analysis of activity data.
(DOC)
