# Correction: Heat Loss May Explain Bill Size Differences between Birds Occupying Different Habitats

**DOI:** 10.1371/annotation/7720a3b2-ef08-42e3-818b-6199c3a86b2c

**Published:** 2012-08-03

**Authors:** Russell Greenberg, Viviana Cadena, Raymond M. Danner, Glenn Tattersall

The images for figures 2,3, and 4 were incorrectly switched. The image that appears as Figure 2 should be Figure 4. The image that appears as Figure 3 should be Figure 2, and the image that appears as Figure 4 should be Figure 3. The figure legends appear in the correct order. 

