# Correction: Heat Loss May Explain Bill Size Differences between Birds Occupying Different Habitats

**DOI:** 10.1371/annotation/07dea6b3-c003-432b-9438-7f3543ab9311

**Published:** 2012-08-10

**Authors:** Russell Greenberg, Viviana Cadena, Raymond M. Danner, Glenn J. Tattersall

The last author's name was misspelled. The correct name is: Glenn J. Tattersall. The correct citation is: Greenberg R, Cadena V, Danner RM, Tattersall GJ (2012) Heat Loss May Explain Bill Size Differences between Birds Occupying Different Habitats. PLoS ONE 7(7): e40933. doi:10.1371/journal.pone.0040933 

