# Supplementary material for: Heat Loss May Explain Bill Size Differences between Birds Occupying Different Habitats
Source: PLoS One. 2012 Jul 25;7(7):e40933. doi: 10.1371/journal.pone.0040933 (PMC3405045; doi:10.1371/journal.pone.0040933)
Supplement: Table S1 — Linear mixed models describing the surface temperature of the bill (Tbill). (DOC) [file pone.0040933.s002.doc]

Table S1. Linear mixed models describing the surface temperature of the bill (*T_bill_*).

| **Models** | **K** | **AICc** | **∆AICc** | **AICc weight** |
| --- | --- | --- | --- | --- |
| SSP + *T_a_* | 6 | 861.232 | 0 | 0.208 |
| SSP *+T_a_* + *T_a_*^2^ | 7 | 861.571 | 0.339 | 0.176 |
| SSP * *T_a_* | 7 | 861.730 | 0.498 | 0.162 |
| *T_a_* | 5 | 862.071 | 0.839 | 0.137 |
| *T_a_* + *T_a_*^2^ | 6 | 862.371 | 1.139 | 0.118 |
| SSP + *T_a_* + *T_a_*^2^ + *T_a_*^3^ | 8 | 863.079 | 1.847 | 0.083 |
| *T_a_* + *T_a_*^2^ + *T_a_*^3^ | 7 | 863.884 | 2.652 | 0.055 |
| SSP * *T_a_* + SSP * *T_a_*^2^ | 9 | 864.018 | 2.786 | 0.052 |
| SSP * *T_a_* + SSP * *T_a_*^2^ + SSP * *T_a_*^3^ | 11 | 867.675 | 6.443 | 8.315E-03 |
| 1 | 4 | 1393.724 | 532.492 | 4.896E-117 |
| SSP | 5 | 1394.370 | 533.138 | 3.544E-117 |

Individual is a random effect and square root of activity is a fixed effect in each model. 1 = neither SSP nor temperature terms are included.
