# Supplementary material for: Heat Loss May Explain Bill Size Differences between Birds Occupying Different Habitats
Source: PLoS One. 2012 Jul 25;7(7):e40933. doi: 10.1371/journal.pone.0040933 (PMC3405045; doi:10.1371/journal.pone.0040933)
Supplement: Table S2 — Linear mixed models describing surface temperature of the bill base (Tbase). (DOC) [file pone.0040933.s003.doc]

Table S2. Linear mixed models describing surface temperature of the bill base (*T_base_*).

| **Models** | **K** | **AICc** | **∆AICc** | **AICc weight** |
| --- | --- | --- | --- | --- |
| SSP * *T_a_* | 7 | 860.717 | 0 | 0.720 |
| SSP * *T_a_* + SSP * *T_a_*^2^ | 9 | 863.740 | 3.024 | 0.159 |
| SSP + *T_a_* | 6 | 866.368 | 5.651 | 0.043 |
| SSP + *T_a_* + *T_a_*^2^ | 7 | 867.625 | 6.908 | 0.023 |
| SSP * *T_a_* + SSP * *T_a_*^2^ + SSP * *T_a_*^3^ | 11 | 867.663 | 6.946 | 0.022 |
| *T_a_* | 5 | 868.604 | 7.887 | 0.014 |
| SSP + *T_a_* + *T_a_*^2^ + *T_a_*^3^ | 8 | 869.511 | 8.794 | 8.865E-03 |
| *T_a_* + *T_a_*^2^ | 6 | 869.764 | 9.047 | 7.810E-03 |
| *T_a_* + *T_a_*^2^ + *T_a_*^3^ | 7 | 871.650 | 10.933 | 3.042E-03 |
| SSP | 5 | 1319.688 | 458.972 | 1.559E-100 |
| 1 | 4 | 1321.402 | 460.686 | 6.616E-101 |

Individual is a random effect and square root of activity is a fixed effect in each model. 1 = neither SSP nor temperature terms are included.
