# Supplementary material for: Heat Loss May Explain Bill Size Differences between Birds Occupying Different Habitats
Source: PLoS One. 2012 Jul 25;7(7):e40933. doi: 10.1371/journal.pone.0040933 (PMC3405045; doi:10.1371/journal.pone.0040933)
Supplement: Table S3 — Linear mixed models describing heat loss through the bill (Qbill). (DOC) [file pone.0040933.s004.doc]

Table S3. Linear mixed models describing heat loss through the bill (*Q_bill_*).

| **Models** | **K** | **AICc** | **∆AICc** | **AICc weight** |
| --- | --- | --- | --- | --- |
| SSP * *T_a_* | 7 | -1578.213 | 0 | 0.462 |
| SSP + *T_a_* | 6 | -1576.144 | 2.070 | 0.164 |
| SSP * *T_a_* + SSP * *T_a_*^2^ | 9 | -1575.855 | 2.358 | 0.142 |
| SSP + *T_a_* + *T_a_*^2^ | 7 | -1575.697 | 2.517 | 0.131 |
| SSP + *T_a_* + *T_a_*^2^ + *T_a_*^3^ | 8 | -1574.063 | 4.150 | 0.058 |
| SSP * *T_a_* + SSP * *T_a_*^2^ + SSP * *T_a_*^3^ | 11 | -1572.134 | 6.080 | 0.022 |
| *T_a_* | 5 | -1570.376 | 7.838 | 9.181E-03 |
| *T_a_* + *T_a_*^2^ | 6 | -1569.979 | 8.234 | 7.529E-03 |
| *T_a_* + *T_a_*^2^ + *T_a_*^3^ | 7 | -1568.325 | 9.888 | 3.293E-03 |
| SSP | 5 | -1475.668 | 102.545 | 2.497E-23 |
| 1 | 4 | -1470.258 | 107.956 | 1.669E-24 |

Individual is a random effect and square root of activity is a fixed effect in each model. 1 = neither SSP nor temperature terms are included.
