# Supplementary material for: Heat Loss May Explain Bill Size Differences between Birds Occupying Different Habitats
Source: PLoS One. 2012 Jul 25;7(7):e40933. doi: 10.1371/journal.pone.0040933 (PMC3405045; doi:10.1371/journal.pone.0040933)
Supplement: Table S4 — Linear mixed models describing heat loss through the bill as a percent of heat of heat lost through all body surfaces (percent Qbill). (DOC) [file pone.0040933.s005.doc]

Table S4. Linear mixed models describing heat loss through the bill as a percent of heat of heat lost through all body surfaces (percent *Q_bill_*).

| **Models** | **K** | **AICc** | **∆AICc** | **AICc weight** |
| --- | --- | --- | --- | --- |
| SSP + *T_a_* + *T_a_*^2^ | 7 | 784.714 | 0 | 0.358 |
| SSP + *T_a_* | 6 | 785.129 | 0.416 | 0.291 |
| SSP * *T_a_* | 7 | 786.828 | 2.114 | 0.124 |
| SSP + *T_a_* + *T_a_*^2^ + *T_a_*^3^ | 8 | 786.860 | 2.147 | 0.122 |
| SSP * *T_a_* + SSP * *T_a_*^2^ | 9 | 787.644 | 2.930 | 0.083 |
| SSP * *T_a_* + SSP * *T_a_*^2^ + SSP * *T_a_*^3^ | 11 | 791.936 | 7.222 | 0.010 |
| *T_a_* + *T_a_*^2^ | 6 | 793.148 | 8.435 | 5.279E-03 |
| *T_a_* | 5 | 793.579 | 8.866 | 4.256E-03 |
| *T_a_* + *T_a_*^2^ + *T_a_*^3^ | 7 | 795.269 | 10.555 | 1.829E-03 |
| SSP | 5 | 815.845 | 31.131 | 6.225E-08 |
| 1 | 4 | 823.817 | 39.104 | 1.156E-09 |

Individual is a random effect and square root of activity is a fixed effect in each model. 1 = neither SSP nor temperature terms are included.
