# Supplementary material for: Heat Loss May Explain Bill Size Differences between Birds Occupying Different Habitats
Source: PLoS One. 2012 Jul 25;7(7):e40933. doi: 10.1371/journal.pone.0040933 (PMC3405045; doi:10.1371/journal.pone.0040933)
Supplement: Table S5 — Linear mixed models describing the surface temperature of the body (Tbody). (DOC) [file pone.0040933.s006.doc]

Table S5. Linear mixed models describing the surface temperature of the body (*T_body_*).

| **Models** | **K** | **AICc** | **∆AICc** | **AICc weight** |
| --- | --- | --- | --- | --- |
| SSP * *T_a_* | 7 | 422.177 | 0 | 0.341 |
| *T_a_* | 5 | 422.831 | 0.654 | 0.246 |
| *T_a_* + *T_a_*^2^ | 6 | 424.341 | 2.164 | 0.115 |
| SSP + *T_a_* | 6 | 424.861 | 2.685 | 0.089 |
| SSP * *T_a_* + SSP * *T_a_*^2^ | 9 | 425.796 | 3.619 | 0.056 |
| *T_a_* + *T_a_*^2^ + *T_a_*^3^ | 7 | 425.833 | 3.657 | 0.055 |
| SSP + *T_a_* + *T_a_*^2^ | 7 | 426.393 | 4.216 | 0.041 |
| SSP * *T_a_* + SSP * *T_a_*^2^ + SSP * *T_a_*^3^ | 11 | 426.584 | 4.407 | 0.038 |
| SSP + *T_a_* + *T_a_*^2^ + *T_a_*^3^ | 8 | 427.908 | 5.732 | 0.019 |
| 1 | 4 | 1427.852 | 1005.675 | 1.422E-219 |
| SSP | 5 | 1429.933 | 1007.756 | 5.024E-220 |

Individual is a random effect and square root of activity is a fixed effect in each model.
