# Supplementary material for: Heat Loss May Explain Bill Size Differences between Birds Occupying Different Habitats
Source: PLoS One. 2012 Jul 25;7(7):e40933. doi: 10.1371/journal.pone.0040933 (PMC3405045; doi:10.1371/journal.pone.0040933)
Supplement: Table S6 — Linear mixed models describing the surface temperature of the legs (Tlegs). (DOC) [file pone.0040933.s007.doc]

Table S6. Linear mixed models describing the surface temperature of the legs (*T_legs_*).

| **Models** | **K** | **AICc** | **∆AICc** | **AICc weight** |
| --- | --- | --- | --- | --- |
| SSP + *T_a_* + *T_a_*^2^ + *T_a_*^3^ | 8 | 1031.015 | 0 | 0.491 |
| *T_a_* + *T_a_*^2^ + *T_a_*^3^ | 7 | 1031.410 | 0.394 | 0.404 |
| SSP * *T_a_* + SSP * *T_a_*^2^ + SSP * *T_a_*^3^ | 11 | 1034.627 | 3.612 | 0.081 |
| SSP + *T_a_* + *T_a_*^2^ | 7 | 1038.805 | 7.790 | 0.010 |
| *T_a_* + *T_a_*^2^ | 6 | 1039.358 | 8.343 | 7.585E-03 |
| SSP * *T_a_* + SSP * *T_a_*^2^ | 9 | 1039.632 | 8.617 | 6.612E-03 |
| SSP * *T_a_* | 7 | 1061.024 | 30.008 | 1.497E-07 |
| SSP + *T_a_* | 6 | 1061.654 | 30.638 | 1.093E-07 |
| *T_a_* | 5 | 1061.883 | 30.868 | 9.741E-08 |
| 1 | 4 | 1445.167 | 414.151 | 5.750E-91 |
| SSP | 5 | 1446.224 | 415.208 | 3.390E-91 |

Individual is a random effect and square root of activity is a fixed effect in each model. 1 = neither SSP nor temperature terms are included.
