# Supplementary material for: Heat Loss May Explain Bill Size Differences between Birds Occupying Different Habitats
Source: PLoS One. 2012 Jul 25;7(7):e40933. doi: 10.1371/journal.pone.0040933 (PMC3405045; doi:10.1371/journal.pone.0040933)
Supplement: Table S7 — Linear mixed models describing relative humidity. (DOC) [file pone.0040933.s008.doc]

Table S7. Linear mixed models describing relative humidity.

| **Model** | **k** | **AICc** | **ΔAICc** | **AICc weight** |
| --- | --- | --- | --- | --- |
| *T_a_* | 4 | 1273.854 | 0 | 0.648 |
| *T_a_* + SSP | 5 | 1275.733 | 1.879 | 0.253 |
| *T_a_* * SSP | 6 | 1277.618 | 3.764 | 0.099 |
| 1 | 3 | 1688.228 | 414.374 | 6.78E-91 |
| SSP | 4 | 1690.235 | 416.381 | 2.49E-91 |

Individual trial is a random effect in each model. SSP=subspecies, *T_a_*=ambient temperature, 1=intercept only.
