# Supplementary material for: Heat Loss May Explain Bill Size Differences between Birds Occupying Different Habitats
Source: PLoS One. 2012 Jul 25;7(7):e40933. doi: 10.1371/journal.pone.0040933 (PMC3405045; doi:10.1371/journal.pone.0040933)
Supplement: Text S1 — Analysis of activity data. (DOC) [file pone.0040933.s009.doc]

Text S1. Analysis of activity data.

Because activity might affect body temperature and heat loss, we tested if activity was related to *T_a_*. We quantified activity as the number of hops per 5-minute trial, which were observed in the thermal imaging videos. Activity data were overdispersed though not zero-inflated. We first fit a generalized linear mixed model with a poisson error distribution using package lme4 [1]. Fixed terms included *T_a_*, *T_a_*^2^, and SSP and individual bird was a random effect. Comparison of the sum of squared Pearson residuals to the residual degrees of freedom [2] indicated that the data were overdispersed (χ^2^=2544, p<0.00001). Because 39% of the values were 0s, we tested for zero-inflation by fitting a zero-altered model using MCMCglmm [3] with 130,000 iterations, thinning interval of 10, and a burn in of 3000. MCMCp values for the zero-altered parameters of *T_a_* (0.777) and *T_a_*^2^ (0.765) indicated that the data were not zero-inflated [3]. To account for overdispersion, we fit the data with a model using the quasi-poisson distribution with package MASS [4]. Correlograms based on the quasi-poisson model indicated that the activity data were not autocorrelated. The relationship between activity and *T_a_* was quadratic (Appendix Figure 1, *T_a_*: β=-0.338 ± 0.068 s.e., p<0.0001, *T_a_*^2^: β=0.0063 ± 0.0014 s.e., p<0.0001), though SSP did not have a significant effect (SSPE: β=-0.328 ± 0.83 s.e., p=0.6986).

Text S1 References

1. Bates D, Maechler M, Bolker B (2011) lme4: Linear mixed-effects models using S4 classes. R package version 0.999375-42. http://CRAN.R-project.org/package=lme4
2. Bolker BM, Brooks ME, Clark CJ, Geange SW, Poulsen JR, Stevens MHH, White J-SS (2009) Generalized linear mixed models: a practical guide for ecology and evolution. Trends in Ecology and Evolution 24:127-135.
3. Hadfield JD (2010) MCMC Methods for Multi-Response Generalized Linear Mixed Models: The MCMCglmm R Package. Journal of Statistical Software, 33(2), 1-22. http://www.jstatsoft.org/v33/i02/.
4. Venables WN, Ripley BD (2002) Modern Applied Statistics with S. Fourth Edition. Springer, New York. ISBN 0-387-95457-0.
